# Natural products targeting ferroptosis in cancer: molecular mechanisms and applications

**DOI:** 10.3389/fonc.2025.1588668

**Published:** 2025-09-09

**Authors:** Xin Ye, Xiaoli Ju

**Affiliations:** ^1^ Department of Pathology, Danyang Hospital of Traditional Chinese Medicine, Zhenjiang, Jiangsu, China; ^2^ Department of Pathology, School of Medicine, Jiangsu University, Zhenjiang, Jiangsu, China

**Keywords:** cancer, molecular mechanisms, ferroptosis, natural products, applications

## Abstract

Ferroptosis is a novel class of programmed cell death that is mainly dependent on intracellular iron accumulation and lipid peroxidation. Ferroptosis is closely related to a variety of human diseases, especially different kinds of cancer. Several small molecule inducers have been developed to induce ferroptosis in tumor cells, some of which have been used in clinical studies. However, these chemical small molecules have toxic effects that limit its wide application. Natural products, however, have a natural advantage in cancer therapy due to their low toxicity and side effects. Some natural products have been found to inhibit tumor growth by inducing ferroptosis in tumor cells. In this review, we reviewed the molecular mechanism of ferroptosis and how natural products targeting ferroptosis signaling pathways affect tumor growth. We also analyzed the application of various natural products such as flavonoids, terpenoids, and alkaloids in inducing ferroptosis in tumor cells. This review will assist in the future discovery and study of more natural product inducers that can induce ferroptosis in tumor cells, and ultimately provide insights into identifying natural products that can be applied to clinical applications.

## Introduction

The goal of tumor therapy is to remove tumor cells. Therefore, inducing tumor cell death is an attractive therapeutic target in cancer therapy. Currently, many drugs inhibit tumor growth by inducing programmed cell death (PCD) in tumor cells. These PCD include apoptosis, necroptosis, pyroptosis, autophagy-dependent cell death (ADCD) and the newly discovered ferroptosis (1). Many of these early anticancer drugs inhibit tumor growth by inducing tumor cell apoptosis. However, due to the heterogeneity of tumor cells and tumor resistance to apoptosis, many drugs that induce apoptosis may develop resistance and thus do not achieve the desired therapeutic effect. Therefore, there is an urgent need to develop new drugs that inhibit tumor growth by inducing other types of programmed cell death. Many small molecules have been reported, such as the small molecule drugs emodin (2–4), shikonin (5, 6), and tanshinol A (7), which target necroptosis, and the small molecules berberine, fluoxetine, and ABTL081, which target ADCD.

The concept of ferroptosis was first proposed by Dixon et al. in 2012 (8). It’s a type of iron-dependent programmed cell death caused by an imbalance of intracellular reactive oxygen species (ROS). Increasing evidence suggests that ferroptosis is closely related to the tumorigenesis and therapeutic efficacy of various tumors. Key proteins on the ferroptosis-related signaling pathway are expected to be new targets for cancer therapy. Induction of ferroptosis can reverse anticancer drug resistance, while inhibition of ferroptosis can block specific death processes. Various inducers and inhibitors have been developed for key proteins of the ferroptosis signaling pathway, inducers such as Erastin, MEII, PE, AE, SAS, Sorafenib and inhibitors such as Fer-1, CPX and DFO (9).

On the other hand, many chemotherapy drugs have strong side effects in cancer treatment. Therefore, there is an urgent need to develop drugs with fewer side effects to achieve better treatment outcomes. Natural products refer to compounds extracted from natural sources such as plants, microorganisms, and animals. Natural products usually have complex structures and specific biosynthetic pathways, while chemical small molecules are relatively small organic compounds prepared by chemical synthesis methods, with relatively simple structures and not necessarily naturally produced by living organisms. They have a range of unique advantages in cancer treatment, including abundant sources, low toxicity and side effects, multiple targets and the ability to overcome drug resistance. Many natural products have been used in the clinic, such as paclitaxel, camptothecin and doxorubicin, which have demonstrated their potential and efficacy in the treatment of a wide range of cancers in clinical applications (10). However, the number of these discovered natural products is limited, and many of them inhibit tumor growth by inducing apoptosis. Ferroptosis is a new type of cell death pathway that offers unparalleled advantages compared to traditional apoptosis and necrosis, such as selectively killing tumor cells, involving multiple pathways and reversing drug resistance. Therefore, inhibition of tumor growth by ferroptosis inducers, especially natural product inducers, will provide new therapeutic options for cancer patients, improve therapeutic efficacy, reduce side effects and improve patients’ quality of life.

## Overview and molecular mechanisms of ferroptosis

In 2003, Sonam Dolma et al. first discovered that a new compound, erastin, could selectively kill tumor cells expressing ST and mutant RAS, but erastin-induced cell death did not show apoptotic features and could not be inhibited by caspase inhibitors. Therefore, it is suggested that erastin-induced cell death was presumed to be a completely new form of death (11). Subsequently, Yang and Yagoda et al. found that this form of death could be inhibited by iron chelators. In 2012, Dixon et al. officially named this type of death as ferroptosis (8). Ferroptosis is characterized by the accumulation of iron and a significant increase in lipid peroxidation. This type of cell death is different from traditional forms of death such as apoptosis and necrosis. It has unique biological and molecular characteristics. During ferroptosis, cell rupture does not occur. Morphological features include an increase in the density of mitochondrial membranes, a decrease in volume, a reduction in mitochondrial cristae, a decrease in cristae density and rupture of the mitochondrial outer membrane, but the nuclear morphology is normal but lacks chromatin condensation (12).

**Figure 1:**
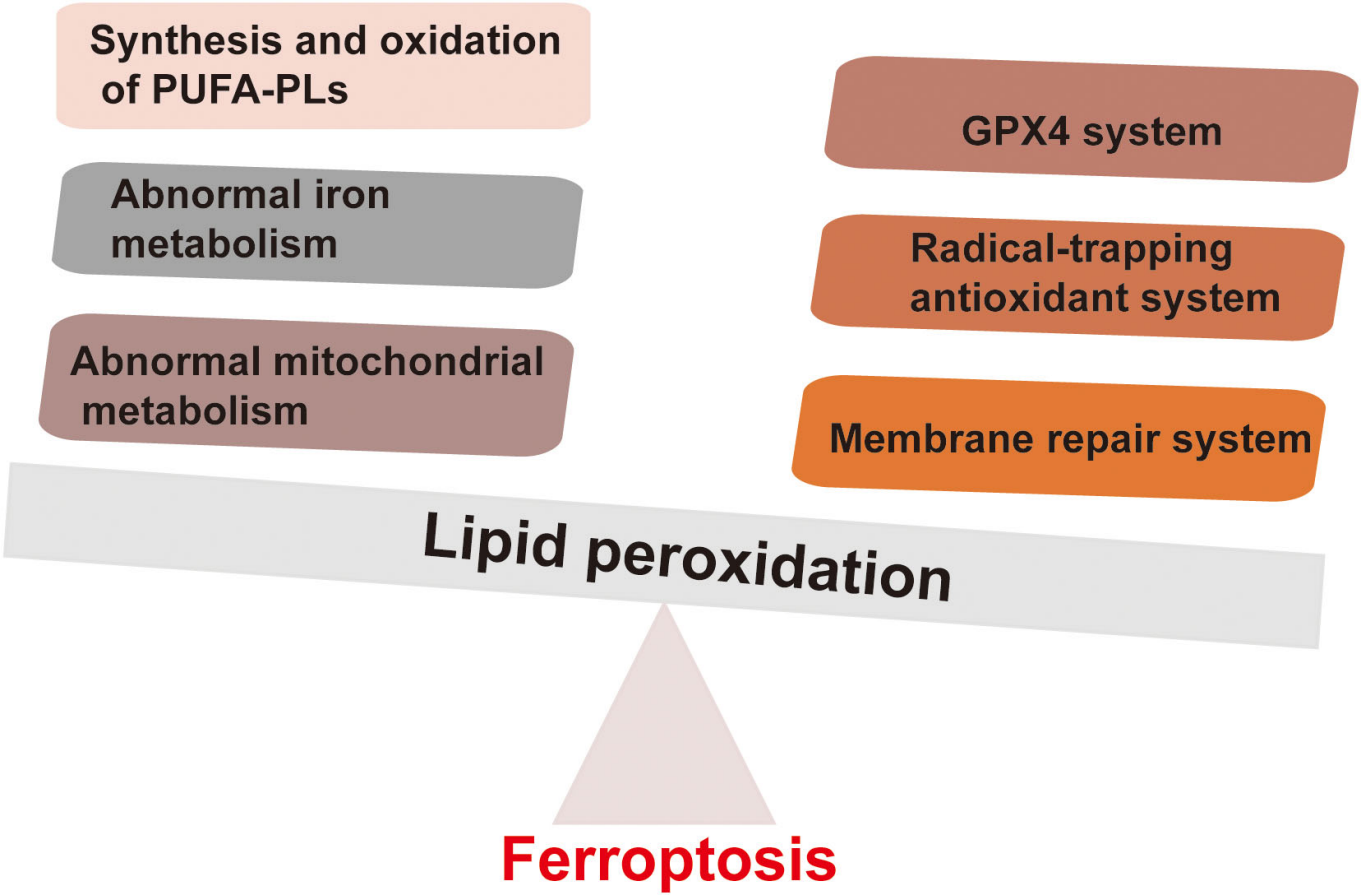
Factors driving ferroptosis and defense mechanisms of ferroptosis. The main factors driving ferroptosis mainly include the synthesis and oxidation of PUFA-PLs, abnormal iron metabolism, and abnormal mitochondrial metabolism. The defense mechanisms against ferroptosis mainly involve the cell antioxidant system that neutralizes lipid peroxides. These antioxidant systems include the GPX4 system, free radical scavenging antioxidant systems (such as FSP1-COQH2 system, DHODH-CoQH2 system, and GCH1-BH4 system).

The main trigger of ferroptosis in cells is the excessive accumulation of lipid peroxides. This is reflected in the disrupts the balance between the execution of ferroptosis and the defense system of the cell, thereby inducing cell ferroptosis. The factors that drive ferroptosis are Polyunsaturated Fatty Acid – Phospholipids (PUFA-PLs) synthesis and oxidation, abnormal iron metabolism, and mitochondrial metabolism abnormalities. The defense mechanism against ferroptosis is mainly the cellular antioxidant system that neutralizes lipid peroxides. These antioxidant systems include the GPX4 system, free radical scavenging antioxidant systems (such as the FSP1-COQH2 system, DHODH-CoQH2 system, and GCH1-BH4 system), and membrane repair systems (**Figure 1**). When the promotion of ferroptosis execution exceeds the cellular defense system, the accumulation of lipid peroxides can induce cell ferroptosis (12, 13) Many ferroptosis inducers have now been designed based on the characteristics of the ferroptosis signaling pathway. Most of them are small molecule compounds and some compounds such as SRF and SAS have been used in the clinic (9).

**Figure 2:**
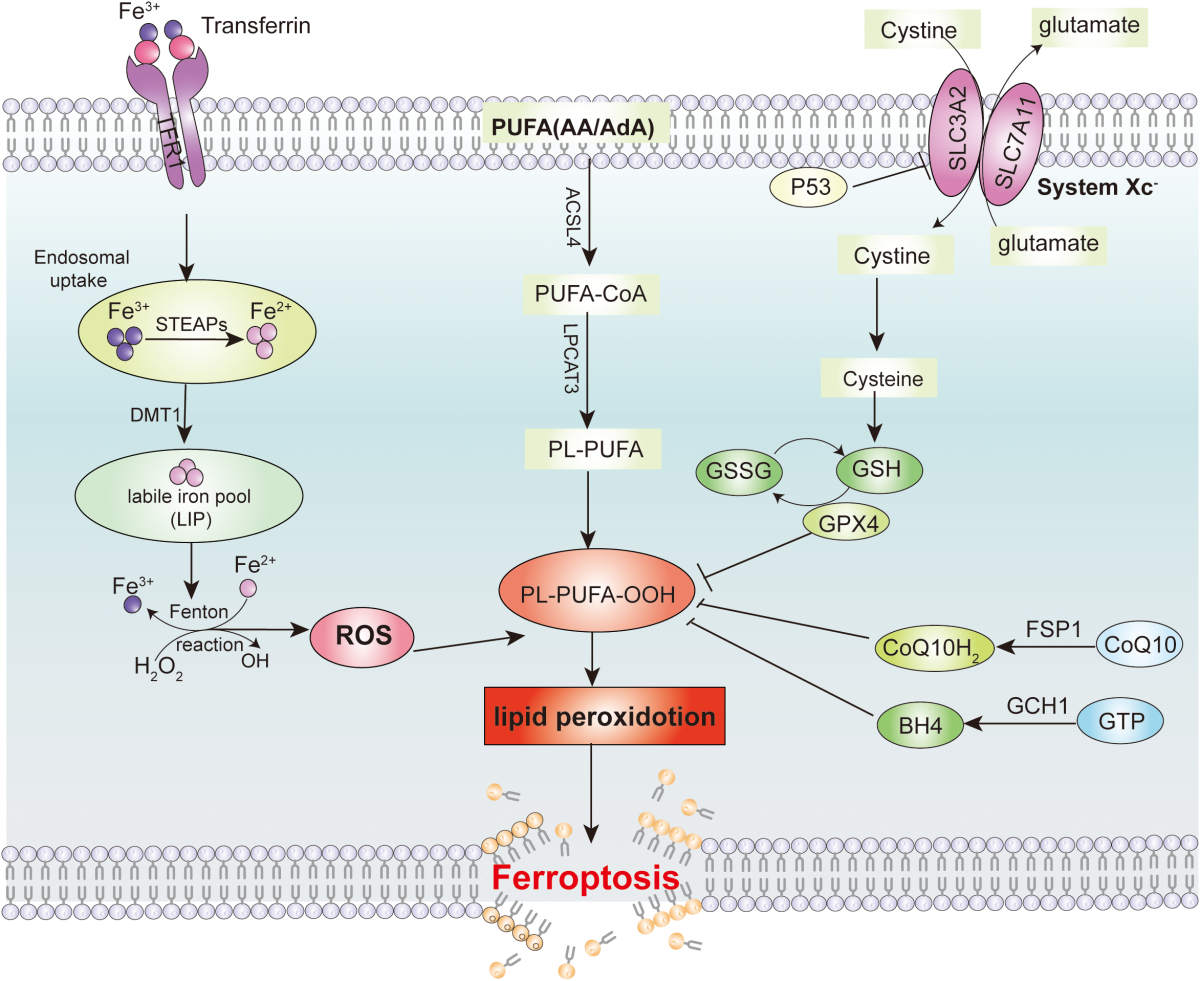
Signaling pathway for inducing ferroptosis in tumor cells. Intracellular iron metabolism and lipid peroxides are the main reasons that induce ferroptosis in tumor cells. The major cellular defense system to avoid ferroptosis are the System Xc^-^/GSH/GPX4 axis, the CoQ/FSP1 axis and the GCH1-BH4 defense system.

Ferroptosis is a form of iron-dependent programmed cell death, and its molecular mechanisms involve multiple signaling pathways. The System Xc^−^-GSH-GPX4 pathway is the core regulatory pathway of ferroptosis. During the process of ferroptosis, the process initially begins with the initiation phase, where the intracellular iron content increases due to enhanced iron uptake mediated by the transferrin receptor (TfR) or increased ferritinophagy. Meanwhile, the function of system Xc^−^ is impaired, leading to a decrease in intracellular glutathione synthesis and a reduction in the activity of glutathione peroxidase 4 (GPX4). Subsequently, in the progression phase, the reduced levels of glutathione and decreased GPX4 activity prevent the timely reduction of lipid peroxides, exacerbating lipid peroxidation reactions and generating a large amount of lipid reactive oxygen species (ROS). ROS directly damage the cell membrane and further promote iron release and mitochondrial dysfunction, creating a vicious cycle. Finally, in the effector phase, mitochondria suffer damage and dysfunction, characterized by a decrease in size, increased membrane density, and a reduction or even disappearance of cristae. Cellular energy metabolism is disrupted, and lipid peroxidation products accumulate in large amounts within the cell. Ultimately, the integrity of the cell membrane is compromised, leading to cell death (14). (**Figure 2**). In addition, NCOA4-mediated ferritin degradation, also known as ferritinophagy, increases the intracellular iron ion levels, promotes lipid peroxidation, and triggers ferroptosis (15). Recent studies have also found that USP13 can promote the transition of ferroptosis to autophagy in tumor cells by activating the NFE2L2/NRF2-SQSTM1/p62-KEAP1 axis in a KRAS signaling pathway-dependent manner (16).

Ferroptosis has emerged as a promising target for cancer therapy (17). However, many small molecule compounds have shown drawbacks such as poor water solubility and targeting ability. Therefore, it is necessary to discover more molecules to induce tumor cell death by other routes. Among them, inducing tumor cell ferroptosis through natural products is a feasible approach.

## Natural products targeting the iron death signaling pathway affect tumor growth

Based on the characteristics of the ferroptosis signaling pathway, natural products that can induce ferroptosis mainly include the following types: 1. Class I that inhibits the system Xc^-^. 2. Class II that inhibits or degrades GPX4. 3. Class III that depletes coenzyme Q10 (18). 4. Class IV that induces lipid peroxidation through iron or PUFA overload (19). These natural products target different targets of the ferroptosis signaling pathways, and ultimately induce ferroptosis in tumor cells to inhibit tumor growth.

## Natural products inhibit system Xc^-^ induce ferroptosis

**Figure 3:**
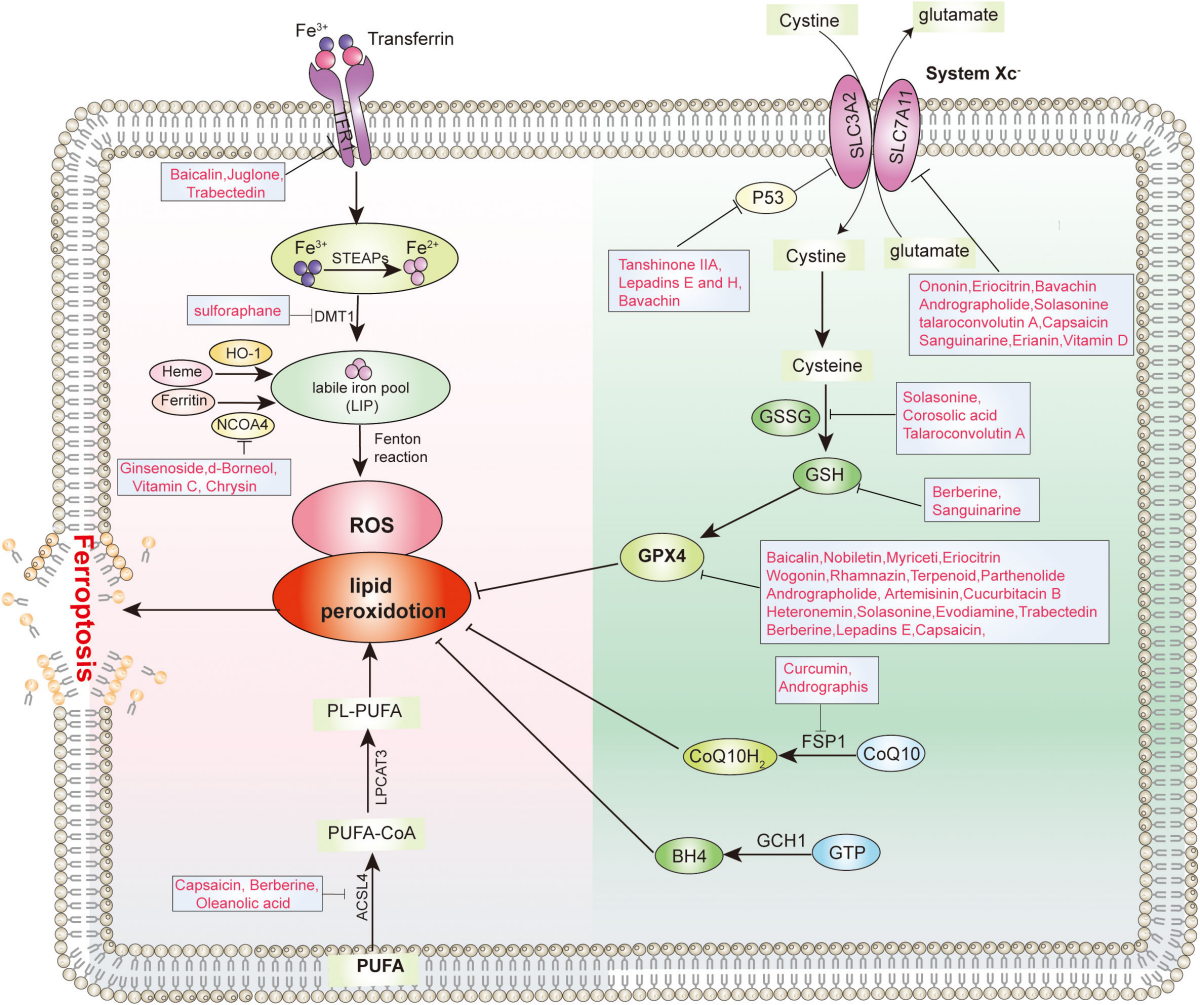
Molecular targets of various types of natural products inhibit tumor ferroptosis.

System Xc^-^ is an important component of the antioxidant system in cells, mainly distributed in the phospholipid bilayer. System Xc^-^ is composed of two subunits, a heterodimer consisting of a heavy chain subunit, SLC3A2, and a light chain subunit, SLC7A11, respectively (20). Cells use system Xc^-^ to uptake cystine into the cell, and the cystine entering the cell is reduced to cysteine, which is an important raw material for synthesizing glutathione (GSH). GSH is an important antioxidant and free radical scavenger, which has a coordinated role with GPX4 to maintain intracellular oxidative balance. By inhibiting System Xc^-^ to limit cysteine intake is the main rate-limiting step in inhibiting glutathione synthesis. Depletion of GSH leads to intracellular redox imbalance, which then leads to intracellular ROS accumulation and ultimately induces ferroptosis. Various natural products targeting System Xc^-^ have been reported to induce ferroptosis, such as kayadiol (21), Bavachin (22), and tanshinone IIA (23) (**Figure 3**).

## Natural products inhibit or degrade GPX4-induced ferroptosis

Glutathione peroxidase 4 (GPX4) is the only enzyme in cells that can reduce lipid peroxides to lipids, and GPX4 plays a crucial role in ferroptosis (24). GPX4 is a selenoprotein that primarily functions to inhibit the formation of lipid peroxides. GPX4 can use GSH as a substrate to specifically catalyze the reduction of lipid peroxides to normal phospholipid molecules. In ferroptosis, when the activity of GPX4 is inhibited, it leads to the accumulation of intracellular peroxides, which in turn causes ferroptosis. RSL3 is one of the most typical inducers of ferroptosis. RSL3 inhibits the activity of GPX4 by covalently binding to the active site of selenocysteine in GPX4, thereby inducing ferroptosis (25). It has been reported that GPX4 is highly expressed in various types of tumors, which may be related to the tumorigenesis (26). Several natural products have been reported to induce ferroptosis by inhibiting GPX4, such as Capsaicin (27), heteronemin (28) and Eriocitrin (29) (**Figure 3**).

## Natural products target iron metabolism to induce ferroptosis

Iron is an important trace element for maintaining the life of living organisms and a cofactor for many biochemical reactions within cells. In ferroptosis, excess intracellular Fe^2+^ can promote the accumulation of lipid ROS through the Fenton reaction, ultimately leading to the induction of ferroptosis. When Fe2^+^ from food is absorbed into the blood, it is oxidized to Fe3^+^. Subsequently, Fe3^+^ binds to transferrin (TF) and is transported to tissues. The transferrin receptor (TFR) on the cell membrane can bind to TF carrying Fe3^+^ and enter the cell through endocytosis. Subsequently, the intracellular Fe^3+^ is reduced to Fe^2+^ by STEAP3 and stored in the labile iron pool (LIP) and in the ferritin consisting of ferritin light chain (FTL) and ferritin heavy chain 1 (FTH1), and small molecule complex GSH. Excess intracellular Fe^2+^ is eliminated from the body mainly through SLC40A1. Due to the instability and high reactivity of Fe2^+^, it participates in the Fenton reaction. The Fenton refers to the reaction where Fe2^+^ reacts with hydrogen peroxide to generate Fe3^+^ and oxygen free radicals. Excessive intracellular iron can generate oxygen free radicals and reactive oxygen through an iron-dependent Fenton reaction, leading to oxidative stress and lipid peroxidation in cells, ultimately inducing cell ferroptosis. Various natural products have been reported to target iron metabolism to induce ferroptosis, such as Baicalin (30), Juglone (31), Trabectedin (32), Vitamin C (33) (**Figure 3**).

## Natural products target lipid metabolism to induce ferroptosis

The formation of lipid peroxides in membrane phospholipids in cells usually leads to ferroptosis. Unsaturated PUFAs, especially arachidonic acid (AA) and adrenoyl acid (AdA), are prone to react with ROS, leading to lipid peroxidation and inducing cell ferroptosis. phosphatidylethanolamines (PEs) containing AA or AdA are key phospholipids that induce ferroptosis. The oxidation of unsaturated PUFAs such as AA is regulated by ACSL4. ACSL4 catalyzes the binding of free AA or AdA with coenzyme A (CoA) to form AA-CoA or AdA-CoA. It is then esterified to membrane AA-PEs by lysophosphatidyltransferase 3 (LPCAT3) and finally undergoes lipid peroxidation catalyzed by the lipoxygenase protein family (LOXs). Therefore, ferroptosis can be inhibited by inhibiting ACSL4 and LPCAT3 or LOXs. Various natural products targeting lipid metabolism have been reported to induce ferroptosis, such as Capsaicin (34), Berberine (35), Oleanolic acid (36) (**Figure 3**).

## Natural products target other pathways to induce iron death

Currently, there are reports showing that there are other pathways and proteins that can affect cell death by ferroptosis. These include coenzyme Q10, nicotinamide adenine dinucleotide phosphate (NADPH), selenium, p53, nuclear factor E2-related factor 2 (NRF2), nuclear factor erythroid 2-related factor 2 (NFE2L2) and Vitamin E.

## Application of natural products in inducing ferroptosis in tumor cells

The active ingredients of natural products originating from the plant kingdom mainly include flavonoids, alkaloids, polysaccharides, volatile oils, quinones, terpenoids, lignans, coumarins, saponins, cardiac glycosides, phenolic acids, amino acids and enzymes. Currently, reports show that most of these types of natural products can induce ferroptosis in tumor cells through various signaling pathways (**Table 1**).

**Table 1:** Various natural products induce iron death in tumor cells.

Category	Compounds	Cancer types	Mechanism	Ref
Flavonoids	bilobetin, isooginkgetin, Ginkgetin	HCT-116, nonsmall cell lung cancer	Enhanced P53 expression, Destruction of Nrf2/HO-1 axis	(38, 85)
	Baicalin	bladder cancer, Osteosarcoma, gastric cancer	Inhibition of FTH1 activity, regulation of the Nrf2/xCT/GPX4 axis, Inhibits TFR1 and others to induce ROS	(30, 40–42)
	Amentoflavone	gastric cancer	Targeting the miR-496/ATF2 axis	(86)
	Nobiletin	multiple myeloma	Induces ROS production by an unknown mechanism that Inhibits NRF2/GPX4	(87, 88)
	4,4’-Dimethoxychalcone	nonsmall cell lung cancer	Keap1/Nrf2/HMOX1 pathway and inhibition FECH,	(89)
	Icariin	prostate cancer	Regulation of miR-7/mTOR/SREBP1 pathway	(90)
	Ononin	Triple-negative breast cancer	Nrf2/SLC7A11 axis	(91)
	Myricetin	gastric cancer	NOX4/NRF2/GPX4 pathway regulation	(92)
	Tiliroside	Pancreatic Cancer, Liver Cancer, Triple Negative Breast Cancer	Targeting calpain-2 to disrupt iron homeostasis, Targeting TBK1, Through the PUFA-PLS pathway	(43–45)
	Quercetin	Breast Cancer, Liver Cancer and gastric cancer	by promoting lysosomal degradation following nuclear translocation of TFEB to activate ferritin, Targeting SLC1A5 Regulates the NRF2/GPX4 Axis	(46–48)
	Eriocitrin	lung adenocarcinoma	Elevated ROS levels, down-regulation of Nrf-2, SLC7A11, and GPX4 expression	(29)
	Isoliquiritigenin	gallbladder cancer	by activating p62-Keap1-Nrf2-HMOX1 signaling	(93)
	Wogonin	Pancreatic cancer	By suppressing the Nrf2/GPX4 axis	(94)
	Lysionotin	colorectal cancer	Promoting Nrf2 Degradation	(95)
	Rhamnazin	hepatocellular carcinoma	inhibiting GPX4 expression	(22)
	Bavachin	Osteosarcoma	STAT3/P53/SLC7A11 Axis	(22, 96)
	Typhaneoside	Leukemia	ROS accumulation	(97)
Terpenoids	Ginsenoside Rh4,	Multiple myeloma (MM), Renal cell carcinoma (RCC)	regulating SIRT2, via the NRF2 Pathway, activating the ROS/p53 signaling pathway	(49–51)
	Ginsenoside Rh5	glioblastoma	Through NR3C1/HSPB1/NCOA4 signaling	(98)
	Ginsenoside Rh3	colorectal cancer	through the Stat3/p53/NRF2 axis	(52)
	Andrographolide	Multiple myeloma	Regulating the P38/Nrf2/HO-1 pathway	(53)
	Abeetic acid	Bladder cancer	activation of the HO-1 pathway	(99)
	Corosolic acid (CA)	liver cancer	upregulating HERPUD1	(100)
	Terpenoid	Hepatocellular Carcinoma	Reduced the expression of GPX4	(28)
	Kayadiol	Extranodal natural killer/T cell lymphoma (NKTCL)	through p53	(21)
	d-Borneol	lung cancer	by promotin NCOA4-mediated ferritinophagy	(101)
	Tagitinin C	Colorectal cancer	through PERK-Nrf2-HO-1 signaling pathway	(102)
	ardisiacrispin B	colon adenocarcinoma	increase in ROS production	(103)
	Oleanolic acid	Cervical cancer	modulation of the ACSL4	(36)
	Parthenolide (PTL)	Hepatocellular carcinoma	Reduce GPX4 expression	(104)
	manoalide	lung cancer	mitochondrial Ca2+ overload induced-FTH1 pathways	(105)
	Andrographolide	Non-Small Cell Lung Cancer, Multiple myeloma	inhibited GPX4 and SLC7A11 expression, block the Nrf2/HO-1 signaling pathway	(53, 54)
	Eupalinolide B	hepatic carcinoma	mediated by endoplasmic reticulum (ER) stress, as well as HO-1 activation.	(106)
	Curcumenol	lung cancer	via lncRNA H19/miR-19b-3p/FTH1 axis	(107)
	Eupaformosanin	triple-negative breast cancer	through ubiquitination of mutant p53	(108)
	Artemisinin and its Derivatives	Head and neck cancer, Glioma, lung cancer, liver cancer	triggering intracellular ROS production, promoting the lysosomal degradation of ferritin and regulating the System Xc-/GPX4 axis.	(56, 57, 63–65)
	Tanshinone IIA	Breast cancer, gastric cancer	destabilizes SLC7A11, through p53-mediated SLC7A11 down-regulation, via the KDM4D/p53 pathway	(23, 109, 110)
	Tagitinin C	colorectal cancer	through PERK-Nrf2-HO-1 signaling pathway	(102)
	Cucurbitacin B	nasopharyngeal cancer	downregulated the expression of GPX4	(111)
	Heteronemin	Hepatocellular Carcinoma	downregulated the expression of GPX4	(28)
Alkaloids	Anisomycin	Hepatocellular Carcinoma	Modulation of the p38 MAPK Pathway	(112)
	Peiminine	breast cancer	through triggering Nrf2 signaling	(113)
	nitidine chloride	multiple myeloma	inhibits PI3K/AKT signaling pathway	(114)
	Chelerythrine	ovarian cancer	through Nrf2	(115)
	nitidine chloride	multiple myeloma	inhibits PI3K/AKT signaling pathway	(114)
	soyauxinium chloride	Melanoma, colon adenocarcinoma	Unknown	(116)
	ungeremine	9 cancers	Unknown	(117)
	Solasonine	hepatoma carcinoma, Pancreatic cancer,	by suppression of GPX4 and GSS, inhibits the TFAP2A/OTUB1 SLC7A11, mitochondrial damage	(66, 67, 118)
	Evodiamine	Bladder Cancer, prostate cancer	Suppression of GPX4, by reducing GPX4 expression,	(68, 69)
	talaroconvolutin A	colorectal cancer, bladder cancer	downregulated SLC7A11 expression, elevated ROS and upregulated transferrin	(119, 120)
	Matrine	Cervical cancer,	through activation of piezo1 channel	(121)
	Cephaeline	lung cancer	by targeting NRF2	(122)
	Trabectedin	Non-small cell lung cancer	via regulation of HIF-1α/IRP1/FE1 and Keap1/Nrf2/GPX4 axis	(32)
	Tomatidine	pancreatic cancer	targets ATF4-dependent signaling	(123)
	Berberine	Nasopharyngeal carcinoma	through System Xc/GSH/GPX4 Axis	(35)
	Solanine	colorectal cancer	through ALOX12B/ADCY4 molecular axis	(124)
	Lepadins E and H	Cervical cancer And melanoma	through the p53-SLC7A11-GPX4 pathway	(125)
	Piperlongumine	various tumors	by Targeting Selenocysteine Residues	(126)
	Capsaicin	Glioblastoma, NSCLC, Hepatocellular carcinoma,	through ACSL4/GPX4 signaling pathways	(27, 34, 70)
	Sanguinarine	Cervical Cancer	involved SLC7A11 down-regulation, GSH depletion,	(127)
	brucine	Hepatocellular Carcinoma	via promotion of hydrogen peroxide and iron	(128)
Phenols	Curcumin	Follicular thyroid cancer, Breast Cancer, osteosarcoma, Colorectal Cancer, breast cancer	increasing the HO-1 expression, by regulating Nrf2/GPX4 signaling, via PI3K/Akt/mTOR or JNK Signaling, by promoting SLC1A5	(18, 71, 129–132)
	Erianin	lung cancer, Bladder Cancer, colorectal cancer, hepatocellular carcinoma	Via Ca2+/CaM signaling, via NRF2 Inactivation, through autophagy-dependent, through the JAK2/STAT3/SLC7A11 pathway	(73–76)
	Gallic acid	hepatocellular carcinoma	via inactivating Wnt/β-catenin signaling pathway	(133)
	Honokiol	ovarian cancer, colon cancer, Acute Myeloid Leukemia	through the regulation of YAP by OTUB2, by regulating GPX4 activity, by Upregulating HMOX1	(134–136)
	Resveratrol	acute myeloid leukemia (AML)	through Hsa-miR-335-5p/NFS1/GPX4 pathway	(137)
	Propofol	non-small cell lung cancer	through the miR-744-5p/miR-615-3p axis	(138)
	6-Gingerol	Lung cancer	via suppression of USP14 expression	(139)
Others	Vitamin C	Pancreatic Cancer, Anaplastic thyroid cancer (ATC)	Activating the AMPK/Nrf2/HMOX1 Pathway	(33, 79)
	Vitamin D	Colorectal Cancer	via SLC7A11 Downregulation	(80)
	Osthole	colorectal cancer	via suppressing AMPK/Akt signaling	(81)
	Withaferin A	hepatocellular carcinoma	via Nrf2-mediated EMT	(82)

## Flavonoids induce ferroptosis in tumor cells

Flavonoids are a class of compounds with a flavone skeleton, widely distributed in certain plants and herbs. Most natural flavonoids exist in the form of glycosides. Among the flavonoids reported to induce ferroptosis in tumor cells are Ginkgetin, Baicalin, Amentoflavone, Nobiletin, and 4,4-Dimethoxychalcone (**Table 1**) (37).

Biflavonoids like bilobetin, isooginkgetin, and ginkgetin from Ginkgo biloba can inhibit MDM2 to boost wild-type P53 expression. This significantly raises ROS levels in colon cancer HCT-116 cells, triggering ferroptosis. Ginkgetin also enhances 5-fluorouracil’s anti-tumor effect in these cells (38). Furthermore, studies have demonstrated that ginkgetin enhances the therapeutic efficacy of cisplatin (DDP) in EGFR wild-type non-small cell lung cancer by inducing ferroptosis via disruption of the Nrf2/HO-1 axis. These findings indicate that ginkgetin not only induces ferroptosis in tumor cells but also potentiates the therapeutic effects of certain chemotherapeutic agents. Nevertheless, further research is required to elucidate the underlying mechanisms.

Baicalin, a flavonoid compound extracted from the roots of *Scutellaria baicalensis*, exhibits significant anti-tumor activity. It induces ferroptosis in tumor cells through multiple mechanisms (39). In bladder cancer and oral squamous cell carcinoma (OSCC), baicalin induces ferroptosis by suppressing the activity of FTH1 (30, 40). In osteosarcoma (OS), it triggers ferroptosis through the regulation of the Nrf2/xCT/GPX4 axis (41). Furthermore, in gastric cancer, baicalin inhibits TFR1 to facilitate ROS-mediated ferroptosis, thereby enhancing the therapeutic efficacy of 5-fluorouracil (42).

Tiliroside, a compound present in various plants, has been demonstrated to inhibit the growth of multiple tumors through ferroptosis, such as triple-negative breast cancer, liver cancer and pancreatic cancer (43–45). In pancreatic cancer, it disrupts iron homeostasis and triggers ferroptosis through direct targeting of calpain-2 (43). In liver cancer, it induces ferroptosis by targeting TBK1 and sensitizes tumors to the chemotherapy drug sorafenib (44). In triple-negative breast cancer, tiliroside induces ferroptosis in TNBC cells through the PUFA-PLS pathway, which is associated with the Nrf2/HO-1 pathway (45). Thus, tiliroside can induce ferroptosis in tumor cells through multiple mechanisms. Quercetin is a natural flavonoid abundant in various plants. In human liver cancer cells, it mediates ferritin degradation through TFEB-dependent lysosomal activation, thereby promoting ferroptosis via iron release and subsequent lipid oxidation (46). A similar mechanism is also found in breast cancer, where quercetin induces ferroptosis by promoting lysosomal degradation of ferritin through TFEB nuclear translocation (47). In gastric cancer, quercetin induces ferroptosis in gastric cancer cells by targeting SLC1A5 and regulating the p-Camk2/p-DRP1 and NRF2/GPX4 axis (48).

## Terpenoids induce ferroptosis in tumor cells

Terpenoids are widely distributed in nature and constitute the main components of fragrances, resins, and pigments in certain plants. As polymers formed by the head-to-tail linkage of isoprene units in various configurations, they exhibit structural diversity. A variety of terpenoids, including Heteronemin, Kayadiol, Corosolic acid, Parthenolide, Curcumenol and Manoalide, have been reported to inhibit tumor growth by inducing ferroptosis in tumor cells (**Table 1**).

Ginsenosides, key bioactive components in ginseng, are triterpene glycosides. Over 40 compounds have been isolated from ginseng roots. Those reported to induce tumor cell ferroptosis include primarily Rh4, Rh3, and Rg5, with Rh4 being the most extensively studied. Notably, Rh4 triggers ferroptosis in malignancies like multiple myeloma (MM) and colorectal cancer (CRC).

In renal cell carcinoma (RCC), Rh4 induces ferroptosis primarily via the NRF2 pathway (49). In multiple myeloma, Rh4 induces ferroptosis mainly through SIRT2 (50). In gastric cancer, it induced ferroptosis through the activation of ROS/p53 signaling pathway and activation of autophagy (51). In glioma, ginsenoside Rg5 inhibits the progression of glioblastoma by activating ferroptosis via the NR3C1/HSPB1/NCOA4 axis (50). Meanwhile, ginsenoside Rh3 (GRh3), a semi-natural product derived from chemical processing, induces both pyroptosis and ferroptosis in CRC cells via the Stat3/p53/NRF2 axis (52).

Andrographolide, the primary bioactive component of *Andrographis paniculata*, is a diterpenoid lactone with potent anticancer activity. In multiple myeloma, it induces ferroptosis in MM cells by activating p38 and subsequently blocking the Nrf2/HO-1 pathway (53). In NSCLC cells, andrographolide downregulates the ferroptosis-related proteins GPX4 and SLC7A11, exacerbates mitochondrial dysfunction, and ultimately triggers ferroptosis (54).

Terpene lactones (NTLs), including γ-lactones and δ-lactones, are a large part of terpenes. Such compounds have a wide range of biological activities. Sesquiterpene lactones, a large group of secondary metabolites predominantly derived from Asteraceae plants, include artemisinin, a sesquiterpene lactone with an endoperoxide bridge extracted from Artemisia annua. Its derivatives comprise artemether (ARM), arteether (ARTE), dihydroartemisinin (DHA), and artesunate (ATS), and various derivatives have been reported to induce ferroptosis in tumor cells (55).

Artemisinin can induce ferroptosis of tumor cells through multiple mechanisms, such as triggering intracellular ROS production, promoting lysosomal degradation of ferritin, and regulating the System Xc-/GPX4 axis to induce ferroptosis (56, 57). Among artemisinin derivatives, DHA has been most extensively studied, with its mechanisms well characterized. DHA is produced by reducing artemisinin with sodium borohydride; compared to the parent compound, it exhibits greater water solubility, a higher metabolic rate, faster absorption, lower cytotoxicity, and reduced drug resistance. Early studies demonstrated that in head and neck cancer, DHA specifically inhibits cancer cell growth by inducing both ferroptosis and apoptosis (58, 59). Subsequently, it was found that DHA induces ferroptosis in glioma cells through the PERK-ATF4-HSPA5-GPX4 pathway (60), with GPX4 identified as a key target of DHA-mediated ferroptosis in glioblastoma (61). In liver cancer, DHA induces hepatocyte ferroptosis by inhibiting ATF4, SLC7A11 or xCT, and also induces ferroptosis in hepatocellular carcinoma by promoting PEBP1/15-LO formation (62). Additionally, other reports suggest DHA triggers ferroptosis in primary liver cancer cells by upregulating CHAC1 expression, which is induced through interactions with unfolded proteins (63). The molecular mechanisms behind this difference still need further in-depth study. In lung cancer, DHA inhibits proliferation and colony formation, increases cell death, and induces ferroptosis in lung cancer cells by inactivating the PRIM2/SLC7A11 axis (64). Subsequently, it was found that DHA not only induces ferroptosis through lipid peroxide (LPO) accumulation but also promotes immunogenic cell death of lung cancer cells, thereby enhancing anti-tumor effects (65).

## Alkaloids induce ferroptosis in tumor cells

Solasonine, a steroidal alkaloid derived from the natural herb Solanum melongena, exhibits potent anticancer activity. In gastric cancer, it induces ferroptosis by inhibiting GPX4 and GSS, thereby elevating lipid ROS levels; this effect can be significantly reversed by ferroptosis inhibitors (66). In pancreatic cancer, by contrast, solasonine activates ferroptosis and suppresses cancer cell progression through inhibition of the TFAP2A/OTUB1/SLC7A11 axis. In lung adenocarcinoma, it triggers tumor cell ferroptosis by disrupting redox balance and causing mitochondrial oxidative stress damage (67) (**Table 1**).

Evodiamine, an alkaloid from Hemerocallis fulva fruits, has antitumor effects. In bladder cancer, it induces ferroptosis mainly by inhibiting GPX4 expression (68). In prostate cancer, Evodiamine acts as a metabolic epigenetic regulator. It increases Sema3A expression to impair angiogenesis and induces ferroptosis by decreasing GPX4 expression. After ferroptosis, HIF1A protein lactylation is inhibited. This blocks lactate-induced angiogenesis, enhances Sema3A transcription and inhibits PD-L1 transcription, boosting antitumor effects (69).

Capsaicinoids, the active components of chili peppers and secondary metabolites, show antitumor activity in various tumors. In glioblastoma, capsaicin induces redox imbalance and ferroptosis in U87-MG and U251 cells primarily via the ACSL4/GPX4 signaling pathway (27). Similar findings were observed in NSCLC, where capsaicin induces ferroptosis mainly by inactivating SLC7A11/GPX4 signaling (34), suggesting its potential as an anticancer agent for NSCLC. Additionally, the synthetic capsaicin analog Arvanil induces high mitochondrial calcium flux, opening the mitochondrial membrane permeability transition pore (mPTP) and triggering ferroptosis in hepatocellular carcinoma (70).

## Phenol induce ferroptosis in tumor cells

Phenolic compounds, formed by hydroxyl groups directly attached to aromatic hydrocarbons, are produced by plants and microorganisms. Curcumin, a yellow pigment extracted from turmeric rhizomes, is an unsaturated polyphenolic compound. It inhibits tumor growth by inducing ferroptosis in various tumor cells, primarily by upregulating HO-1 expression. For example, in Follicular Thyroid Cancer (FTC), HO-1 overexpression activates ferroptosis signaling. Curcumin suppresses FTC growth by inducing ferroptosis through increased HO-1 expression (71) (**Table 1**).

Erianin, extracted from *Dendrobium chrysotoxum* Lindl, shows anti-cancer activity across various cancers, inhibiting tumor growth via ferroptosis in lung, liver, bladder, and colon cancers (72). In lung cancer, it induces ferroptosis and inhibits cell migration through Ca2+/CaM signaling (73). In bladder cancer, Erianin triggers ferroptosis by inactivating NRF2 (74). In colon cancer, it suppresses growth and metastasis via autophagy-dependent ferroptosis in KRAS (75). In liver cancer, Erianin induces ferroptosis by activating JAK2/STAT3 and inhibiting SLC7A11 and GPX4 expression, reducing HCC cell proliferation and invasion (76). Additionally, in kidney cancer, Erianin promotes ferroptosis in cancer stem cells by enhancing ALOX12/p53 mRNA N6-methyladenosine modification (77). It also inhibits lung cancer stemness and improves chemosensitivity by inducing ferroptosis, highlighting its potential in cancer therapy (78).

## Other natural products induce ferroptosis in tumor cells

Other natural products can also induce ferroptosis in tumor cells, including vitamins, coumarins, and steroids (**Table 1**). Vitamin C induces ferroptosis in pancreatic cancer cells and inhibits tumor growth by activating AMPK/Nrf2/HMOX1 (33, 79). while vitamin D promotes ferroptosis in colorectal cancer stem cells by downregulating SLC7A11 (80). Osthole, a natural coumarin from fungi and umbelliferous plants, induces ferroptosis in colon cancer cells by inhibiting the AMPK/Akt/mTOR pathway (81). Withaferin A, a sterol lactone from the medicinal plant Withania somnifera, induces EMT and ferroptosis in liver cancer cells through Nrf2 mediation (82).

Flavonoids, terpenoids, alkaloids and phenols all induce ferroptosis in tumor cells by interfering with the antioxidant system, promoting lipid peroxidation and regulating iron metabolism. However, they differ in their specific mechanisms. Flavonoids induce ferroptosis by releasing iron ions via ferritinophagy and inhibiting antioxidant enzymes. Terpenoids activate ACSL4 to boost lipid peroxidation substrate synthesis and affect mitochondrial function. Alkaloids suppress xCT to block cystine uptake or inhibit the mitochondrial respiratory chain to produce ROS. Phenols directly promote oxidation through the Fenton reaction and inhibit repair of peroxidized phospholipids at high concentrations.

## Conclusion

Ferroptosis is a recently discovered novel mode of programmed death, mainly caused by intracellular iron accumulation and an increase in lipid peroxidation. There are significant differences between ferroptosis and other types of programmed death in terms of cell morphology and molecular mechanisms. Current reports show that ferroptosis is closely related to a variety of human diseases, such as neurological diseases, cardiovascular diseases, infectious diseases and cancer. Inhibiting tumor growth by inducing ferroptosis in various tumor cells has become a popular target in cancer therapy. Some small molecule compounds that can induce tumor cell ferroptosis such as SRF and SAS have been used in clinical studies. However, small molecule compounds have always had side effects in cancer treatment, causing toxic effects on normal cells. Natural products have unique advantages in cancer therapy, such as low toxicity and side effects as well as overcoming drug resistance. Therefore, discovering and studying the use of natural product therapy to induce ferroptosis of tumor cells has important theoretical and practical significance. We systematically analyzed the molecular mechanisms of natural products inducing ferroptosis in tumor cells and the applications of various types of natural products reported in inducing ferroptosis in tumor cells. These analyses provide a theoretical basis and guidance for the discovery and study of more natural product inducers in the future. Although many natural products have been found to induce ferroptosis in tumor cells, however, further research is needed to discover and study the molecular mechanisms and clinical efficacy of these natural products.

Natural products hold certain therapeutic potential in inducing ferroptosis in tumors, but they also face many limitations and challenges. First, tumor cells show significant differences in sensitivity to ferroptosis. Different tumor types vary in their sensitivity to ferroptosis due to differences in metabolic characteristics, gene expression profiles, and genetic mutation patterns. For example, hepatocellular carcinoma, pancreatic cancer, and breast cancer are relatively sensitive to ferroptosis, while some tumor cells may develop tolerance to ferroptosis inducers because they contain high levels of antioxidant enzymes or iron regulatory proteins. In addition, the complexity of the tumor microenvironment can also affect the occurrence of ferroptosis, and genetic mutations that may occur during the process of cell carcinogenesis, such as mutations in the p53 gene, can also change the sensitivity of cells to ferroptosis (83).

Second, the potential side effects of natural products in inducing ferroptosis cannot be ignored. Natural products may have toxic effects on normal tissues, especially at high doses or with long-term use. Some natural products may be photosensitive, thermosensitive, or chemically unstable, which limits their clinical application prospects. Moreover, ferroptosis inducers may increase oxidative stress levels, which in some cases may promote the occurrence and development of tumors. For example, excessive lipid peroxidation can lead to DNA damage, thereby triggering mutations and tumor development. Certain natural products may interact with ferroptosis inducers to produce unknown side effects or enhance toxicity. In summary, although natural products have potential in inducing ferroptosis in tumors, their limitations and challenges should not be overlooked (84).

In future research, we need to focus more on the following issues. First, different types of tumor cells have varying sensitivities to natural products inducing ferroptosis in these tumor cells, and the underlying mechanisms have been unknown. Therefore, future research needs to focus on studying the molecular mechanisms that cause differences in sensitivity. Secondly, many natural products have disadvantages such as poor water solubility, limiting their wider application. Therefore, future studies need to focus on the structurally modifying these natural products that can induce ferroptosis in tumor cells to make them more soluble in water and more easily absorbed by the body. Alternatively, the development of more effective drug delivery vehicles that can deliver these natural products to the tumor site in the human body. Finally, the combination of natural products with chemotherapeutic or targeted drugs for the treatment of tumors can achieve better therapeutic effects, so future research needs to focus on this combination of drug therapy. The in-depth study of the above issues will eventually provide broader prospects for the application of natural products in cancer therapy.

In future research, the induction of ferroptosis by natural products should focus on addressing existing limitations. On the one hand, improving the solubility and bioavailability of natural products through means such as nanotechnology, microemulsion technology, and liposome encapsulation, and developing tumor-targeted nanocarriers or drug delivery systems to enhance their accumulation and efficacy in tumor tissues while reducing toxicity to normal tissues. On the other hand, delving into the molecular mechanisms underlying the sensitivity of tumor cells to ferroptosis, identifying predictive biomarkers, and providing a basis for precision therapy. Research should be committed to the development of combination therapies, integrating ferroptosis inducers with traditional chemotherapy, radiotherapy, targeted therapy, or immunotherapy to enhance therapeutic effects and reduce the resistance associated with monotherapy. For example, combining ferroptosis inducers with radiotherapy can enhance DNA damage induced by radiotherapy, while combining them with immune checkpoint inhibitors can improve the immune microenvironment. In addition, it is necessary to explore the interactions between ferroptosis and other forms of cell death, as well as the regulatory role of the tumor microenvironment in these processes, to provide a more comprehensive strategy for cancer treatment.
